# Multiple Drug Intolerance Syndrome and Arterial Hypertension—Systematic Review and Meta-Analysis

**DOI:** 10.3390/jcm14176218

**Published:** 2025-09-03

**Authors:** Jakub Rusinek, Kinga Tyjas, Wiktoria Ziółek, Marek Rajzer, Katarzyna Stolarz-Skrzypek

**Affiliations:** 1 Doctoral School of Medical and Health Sciences, Jagiellonian University Medical College, 31-538 Krakow, Poland; 2 1st Department of Cardiology, Interventional Electrocardiology and Arterial Hypertension, Jagiellonian University Medical College, Jakubowskiego St. 2, 30-688 Krakow, Poland; 3 Students’ Scientific Group at the 1st Department of Cardiology, Interventional Electrocardiology and Arterial Hypertension, Jagiellonian University Medical College, 30-688 Krakow, Poland

**Keywords:** hypertension, multiple drug intolerance syndrome, adverse drug reaction

## Abstract

**Background:** Arterial hypertension (HT) is one of the most prevalent diseases, causing increased morbidity and mortality. Treatment of HT might be complicated by multiple drug intolerance syndrome (MDIS), defined as intolerance to three or more drug classes. This systematic review and meta-analysis aimed to assess the prevalence of MDIS in patients with hypertension and investigate its impact on disease control. **Materials and Methods:** MEDLINE, Scopus, Web of Science, and Embase were thoroughly searched in June 2024. The data concerning MDIS prevalence, MDIS risk factors, number of adverse drug reactions (ADRs), and types of reactions was extracted. Quality assessment was done with the use of the Newcastle-Ottawa Scale. Meta-analysis was conducted to determine the pooled prevalence of MDIS and ADRs. The study was conducted according to the PRISMA guidelines. **Results:** This review included four studies (2508 patients). The pooled prevalence of MDIS was 10% (95% CI: 4%–19%). Headache was the most frequent ADR reported in the studies (71.47%, 95% CI: 56.5%–84.44%), while pain in other body parts was present in 53.08% of patients. Female sex was found to increase the risk of MDIS in each included study. Other risk factors identified were older age, gastrointestinal disorders, anxiety disorders, panic attacks, and depression. Blood pressure was higher in the MDIS group, and in this group, more patients had uncontrolled hypertension. **Conclusions:** The prevalence of MDIS in the hypertensive population can be considered high. Furthermore, blood pressure control is worse in patients with MDIS. However, this phenomenon is still studied inadequately, and further research is necessary.

## 1. Introduction

Arterial hypertension is one of the most prevalent diseases, simultaneously being a significant public health burden. It is estimated to affect 33% of people between 30 and 79 years of age worldwide [1]. High blood pressure values increase the risk of death due to stroke, ischemic heart disease, and other cardiovascular diseases (CVD) [2]. It also contributes to organ failure through its effects on the microcirculation [3]. Therefore, proper management of hypertension is crucial. According to European Society of Cardiology (ESC) recommendations, blood pressure-lowering treatment should combine both lifestyle changes and pharmacological treatment, starting with low-dose double combination therapy. If tolerance to the current regimen is confirmed, doses can be gradually increased [4].

Drug classes commonly used to reduce blood pressure are angiotensin-converting enzyme inhibitors (ACE-I), angiotensin receptor blockers (ARB), calcium channel blockers (CCB), diuretics, and beta-blockers (BB) [4]. Despite the wide range of antihypertensive medications available, only 21% of hypertensive patients aged 30 to 79 years have had their hypertension controlled [1]. One of the reasons for poor hypertension control is the occurrence of adverse drug reactions (ADRs), resulting in patient non-adherence. Antihypertensive drugs are one of the most frequent drug classes involved in ADRs among adults [5]. Furthermore, since the beginning of the therapy, at least two drugs are prescribed, and even though combination therapy is associated with a lower risk of ADRs compared to a dose increase of one drug [6], a patient is at risk of side effects of both drugs. When a patient reports side effects from at least three different classes of drugs that are chemically unrelated, it is called multiple drug intolerance syndrome (MDIS) [7]. The risk of MDIS increases with age, higher body mass index, multimorbidity, and thus high medication usage. It was also observed to be more prevalent in females [7,8]. There is still little information about MDIS prevalence. In a large study from California, it was present in 2.1% of the assessed general population [7], and in a Polish study, it was found in 8% of hypertensive patients [9]. Although MDIS is not a common entity, it poses a significant problem in modern medicine as it affects patients’ treatment, leading to health complications, non-adherence, and drug discontinuation, resulting in poor hypertension control [7,8].

Thus, the aim of this review and meta-analysis was to summarize the current knowledge about the occurrence of MDIS in patients with hypertension and to investigate its impact on disease control and the severity of complications.

## 2. Materials and Methods

The systematic review and meta-analysis were conducted according to the Preferred Reporting Items for Systematic Reviews and Meta-Analyses (PRISMA) guidelines [10]. The study protocol was registered in PROSPERO (https://www.crd.york.ac.uk (accessed on 28 August 2025)) and given the number CRD42024572340. It was not changed after approval.

### 2.1. Search Strategy

A thorough search of four databases was conducted, including MEDLINE, Scopus, Web of Science, and Embase. The time frame was not specified, so all of the studies published until June 2024 were searched. The following PubMed query was used:

*(“hypertension”[MeSH Terms] OR “hypertension”[All Fields] OR (“arterial”[All Fields] AND “hypertension”[All Fields]) OR “arterial hypertension”[All Fields]) AND (“drug”[All Fields] AND (“intolerabilities”[All Fields] OR “intolerability”[All Fields] OR “intolerable”[All Fields] OR “intolerably”[All Fields] OR “intolerance”[All Fields] OR “intolerances”[All Fields] OR “intolerant”[All Fields] OR “intolerants”[All Fields]))*

Queries for other databases were similar and differed only by the specific syntax requirements. Moreover, two systematic reviews focusing on a similar subject were identified during screening, and their references were also assessed [8,11].

### 2.2. Inclusion and Exclusion Process

All of the appropriate studies assessing MDIS combined with hypertension were included. Inclusion and exclusion criteria were established before abstract screening, and they were as follows.

Inclusion criteria:

1. Full-text original study assessing both drug intolerance and hypertension.
2. Human subjects.
3. At least 18 years of age.
4. Outcomes include MDIS prevalence, type of adverse events or hypertension control.

Exclusion criteria:

1. Other publication types (i.e., meta-analyses, reviews, case reports, conference reports).
2. Specific drug trials.

Initially, the abstract screening was conducted independently by two authors. Subsequently, the full texts were also reviewed independently by two authors. Any disagreements were resolved through discussion.

### 2.3. Data Extraction

Data was extracted from included studies with the use of the predefined spreadsheet. It was done by two authors separately, and the differences were later discussed. The following data was extracted: the name of the first author, title, publication year, study design, number of included patients, number of patients with MDIS, MDIS definition used in the study, number and type of ADRs, drugs or drug classes that caused ADRs, blood pressure control, mean values of systolic blood pressure (SBP) and diastolic blood pressure (DBP), organ damage, and factors associated with MDIS.

We attempted to contact Davies et al. to obtain more detailed information about the prevalence of ADRs, but we have not received any feedback [12].

### 2.4. Quality Assessment

Before final inclusion, the quality of each study was assessed using the Newcastle–Ottawa Scale (NOS) [13]. No study was excluded due to low quality.

### 2.5. Statistical Analysis

The meta-analysis was performed for two outcomes: MDIS prevalence and specific adverse event prevalence. Pooled prevalence was estimated using MetaXL version 5.3. The random-effect model was applied. The I^2^ test was used to assess heterogeneity, with the following interpretation: 0%–40% may be insignificant; 30%–60% may indicate moderate heterogeneity; 50%–90% may indicate substantial heterogeneity; and 75%–100% may represent considerable heterogeneity.

## 3. Results

**Figure 1:**
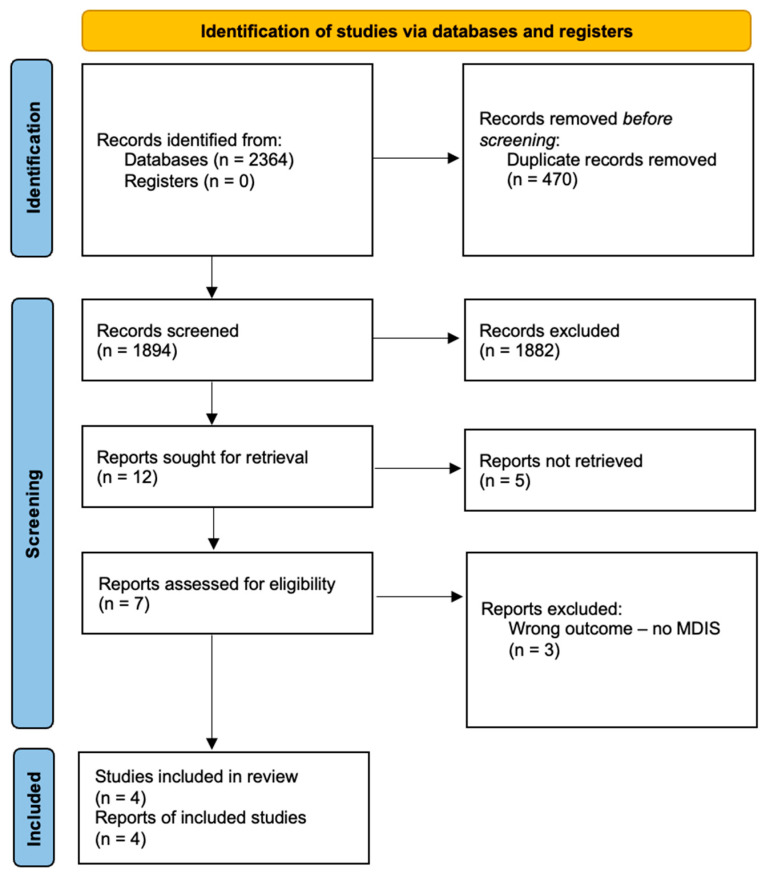
Flowchart of the study. MDIS—multiple drug intolerance syndrome.

In this database search, 1894 unique records were identified (after duplicate removal). Full texts of seven studies were reviewed, and four were eventually included [9,12,14,15]. We did not identify any studies via other methods. Detailed information about the inclusion process can be found in the PRISMA flowchart (Figure 1).

### 3.1. Studies Characteristics

This review included 2508 patients, of whom 240 were categorized as presenting with MDIS. Two studies were conducted in the United Kingdom, one in Nigeria, and one in Poland (Table 1). All studies documented the prevalence of MDIS; three specifically reported on the prevalence of ADRs, three provided information regarding MDIS risk factors, three addressed blood pressure control, and two noted intolerated drugs.

### 3.2. MDIS Prevalence

**Figure 2:**
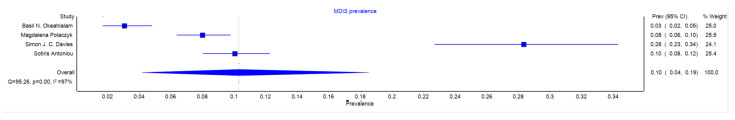
Pooled prevalence of MDIS. CI—confidence interval; MDIS—multiple drug intolerance syndrome [9,12,14,15].

**Figure 3:**
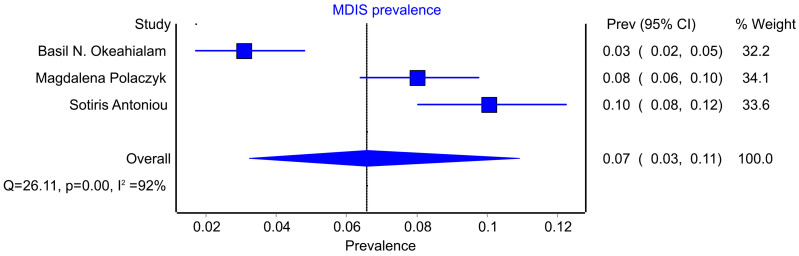
Pooled prevalence of MDIS—sensitivity analysis (Davies et al. excluded). CI—confidence interval; MDIS—multiple drug intolerance syndrome [9,14,15].

The definition of MDIS differed between the studies (Table 1). Each included study reported the prevalence of MDIS, which ranged from 3% to 28% (Figure 2). The calculated pooled prevalence was 10% (95% CI: 4%–19%, I^2^ 97%). To investigate high heterogeneity, another analysis was conducted (Davies et al. study excluded)—pooled prevalence reached 7% (95% CI: 3%–11%) but still with a high level of heterogeneity (I^2^ = 92%) (Figure 3).

### 3.3. Adverse Drug Reactions Prevalence

Three studies reported the occurrence of specific ADRs [9,12,14]. During the ADR prevalence analysis, the ADRs that occurred only once in one study were omitted. Moreover, Davies et al. used another reporting method: they only showed a number of specific ADRs after each medication without specifying how many episodes the MDIS group experienced. We decided to include the data from this study in the analysis as the MDIS group was the majority.

Headache was the most frequent ADR reported in the MDIS studies (71.47%, 95% CI: 56.5%–84.44%). Headache was analyzed separately from pain (in different body parts) which was also quite frequent with a pooled prevalence of 53.08% (95% CI: 0%–100%). Allergic reactions (66.25%, 95% CI: 55.47%–76.25%) and “bad feeling” (59.98%, 95% CI: 33.86%–83.65%) also occurred in the majority of patients, but each was reported in only one (different) study. Detailed information about ADRs prevalence can be found in Table 2.

### 3.4. MDIS Risk Factors

All studies reported risk factors associated with MDIS occurrence [9,12,14,15]. In each study, female sex was found to either increase the odds of having MDIS or to be more frequent in the MDIS group (Table 3).

Furthermore, Polaczyk et al. found gastrointestinal diseases to increase the odds of having MDIS. In a study by Antoniou et al., the analysis was more precise, and specifically, gastroesophageal reflux disease (GERD) was the gastrointestinal tract disease associated with the prevalence of MDIS. When it comes to age, the data is contradictory. Both Davies et al. and Antoniou et al. reported significantly higher age in the MDIS group. Moreover, although Polaczyk et al. did not consider age a significant MDIS risk factor, longer hypertension duration, which was correlated with age, was associated with MDIS. The data about psychiatric comorbidities is also not unambiguous. On the one hand, Polaczyk et al. did not find a significant relationship between the occurrence of MDIS and mental diseases. On the other hand, Antoniou et al. reported a high prevalence of anxiety disorders in the MDIS group (but again without information about occurrence in the group with good treatment tolerance). Furthermore, in a study by Davies et al., they focused in detail on psychiatric comorbidities and found that the prevalence of anxiety disorders, panic attacks, and depression is significantly correlated with the number of intolerance episodes (*p* = 0.040, *p* = 0.008, *p* = 0.005, respectively).

### 3.5. Hypertension Control

Three studies analyzed hypertension control [12,14,15]. However, only two reported the mean SBP and DBP values. Okeahialam found that 80% of the MDIS group had uncontrolled hypertension but without specifying the blood pressure values. MDIS cohort in the study by Antoniou et al. had baseline mean blood pressure of 177/95 mmHg. The SBP was higher than in the reference group (168/96 mmHg), but the information was not included if the difference was statistically significant. The mean BP in the MDIS group in the study by Davies et al. was 168/93 mmHg, which differed from the control group (160/89 mmHg) (*p*-value for SBP = 0.050, *p*-value for DBP = 0.003). Mean BP values were not compared, but they found a significant correlation between the number of intolerated drugs and SBP and DBP.

### 3.6. Intolerated Drugs

Two studies reported the exact number of people from the MDIS group who did not tolerate each drug class [9,15] (Table 4). The drugs most frequently reported as intolerable were antibiotics (46.25%) and analgesics (43.75% or 46.67%). Davies et al. provided only information about the number of intolerance episodes after each drug that might have occurred several times in one person. Therefore, the data might be misleading and was not included in this review. Antoniou et al. reported only a number of drug classes that were not tolerated: by average 5.3 in the MDIS group and 0.2 in the reference group, significantly higher in the MDIS group.

## 4. Discussion

To the best of our knowledge, this is the first systematic review and meta-analysis taking into consideration MDIS, specifically in hypertensive patients. Previously published reviews were conducted without the application of meta-analysis methodology following PRISMA recommendations [8,11]. MDIS is perceived as a rare syndrome, and currently the knowledge about its influence on hypertension treatment is lacking. However, as we proved in this review—the prevalence of MDIS is significant. Thus, it was essential to compile all research addressing MDIS in hypertensive patients, providing a clear overview highlighting knowledge gaps and identifying the patients at risk of MDIS who warrant physicians’ attention.

### 4.1. Definitions of MDIS

In this systematic review, we included four studies that assessed 2508 patients. The most popular definition of MDIS is ADRs to three or more drug classes, and this definition is well-established in the literature [7,16]. However, some authors postulate to require a lack of specific immunological reaction to define MDIS [16]. While in other studies, the existence of an allergy does not disqualify a patient from an MDIS diagnosis [7,17]. Similarly, studies included in this review showed discrepancies in definitions. Firstly, three studies classified MDIS as three or more drug intolerances [9,14,15] while one as two or more drug intolerances [12]. Considering two or more drugs as MDIS might be explained by the year of publication by Davies et al. (2003) when multiple drug allergy syndrome was not distinguished from MDIS, and this definition can also be found in the literature [7,12]. MDIS is a much broader term, taking into consideration many different types of ADRs regardless of mechanisms. On the other hand, drug allergy requires a confirmed IgE-dependent mechanism of hypersensitivity. Secondly, two studies excluded patients with immunological intolerance mechanisms, while two others did not mention this criterion (Table 1). In our opinion, the definition of using three or more drugs regardless of the immunological mechanism should be used in clinical practice in the hypertensive population—as this definition is much more popular in the literature, and it is often impossible to assess the immunological mechanism of intolerance in cardiology clinics [8].

### 4.2. Prevalence of MDIS

When it comes to the prevalence of MDIS in hypertensive patients, our meta-analysis summarized data from all four studies—pooled prevalence reached 10%. This result is in line with research done on the general population in which the MDIS prevalences ranged from 2% to 10% [8,16,18]. However, it is slightly higher than in the previously published review regarding the hypertensive population (6%) [11], but it might be caused by the calculated pooled prevalence in our study, while in the previous review, the result in just one study cohort was reported [17] and the review was not systematic, so the strength of evidence is much lower. Moreover, studies used different MDIS definitions, which may explain not only the much higher MDIS occurrence reported by Davies et al. (as they also included patients with ADRs after two drug classes) [12] but also a high level of heterogeneity. The prevalence presented by Davies et al. is even higher than in another study by Polaczyk et al. reporting the occurrence of intolerance of 2 or more drugs in 16.2% of patients [19]. However, the prevalence was still high (7%) after excluding Davies et al. The exclusion of this study did not explain high heterogeneity. Therefore, data must be interpreted with caution as heterogeneity is probably caused by different study designs and settings.

### 4.3. Adverse Drug Reactions

This is the first review to analyze the prevalence of ADRs in hypertensive patients with MDIS. We found headache to be the most frequent ADR in patients with hypertension and MDIS. Headache was previously described as one of the most frequent ADRs in the general population in the Netherlands, where it was the third most often reported ADR [20]. Patients with hypertension are at risk of headaches as ADRs after ACE-I or CCB, which cause vasodilatation [21]. This was confirmed by a high frequency of intolerance to those two drug classes found in one study [14]. This is particularly important because headaches caused by drugs are hard to distinguish from primary headaches, which, if misdiagnosed, might be treated with several analgesics, potentially leading to medication overuse headaches [20]. Paying more attention to ADRs in clinical setting may help to treat headaches by simply changing the antihypertensive medications.

When it comes to pain, it was hard to establish if it was really an ADR or a symptom of comorbidities. Patients with MDIS most often suffer from several diseases, and many of them may cause pain. Leadley et al. reported in a systematic review that chronic pain prevalence might be as high as 27% in the general population [22]. It was not assessed in this review, but it might be assumed that the prevalence is even higher in the studied population with numerous comorbidities.

Tiredness and “bad feeling” are nonspecific symptoms that were also quite frequent in the MDIS population. A combination of thiazide diuretics and beta-blockers is thought to increase the chance of fatigue and sleepiness [23]—a combination especially popular in older patients with other cardiovascular risk factors. Therefore, it is not apparent if one specific drug caused fatigue in MDIS patients, whether it was caused by the drug combination or, again, by comorbidities.

Although the hypertensive population was studied, ADRs typical for antihypertensive medications were not the most prevalent. For instance, cough was present in 33.94% of patients, while it is a typical ADR caused by ACE-I [24]. A typical ADR caused by CCB (edema) also appeared in the minority of patients—38.86%. Furthermore, each antihypertensive agent may cause hypotension but only 30.00% of patients suffered from this ADR [25]. It might be explained by the distribution of intolerated drugs—antihypertensives were not the most often intolerated drugs in the MDIS population.

### 4.4. MDIS Risk Factors

Acknowledging the risk factors of MDIS plays a crucial role in clinical practice. We identified three risk factors that appeared in each included study—female sex, older age (or longer disease duration), and gastrointestinal tract disorders. Female sex and older age were identified before assessing the general population [7,16,18,26]; moreover, higher body mass index, higher weight, previous hospital admission, and multiple comorbidities were also significant risk factors described in the literature in two large retrospective studies taking into consideration over 50,000 patients [7,18]. On the other hand, as retrospective studies they had several limitations, and their results also have to be interpreted with caution. In our review, we did not observe such a relationship in the hypertensive population. Our data was not consistent in terms of psychiatric comorbidities. Nevertheless, the majority of included studies showed a significant association of MDIS with anxiety and depression, which aligns with the existing research on patients without hypertension [17,27].

### 4.5. Hypertension Control

Hypertension was poorly controlled in patients with MDIS. 80% of patients had uncontrolled hypertension, and mean BP values ranged from 168/93 mmHg to 177/95 mmHg [12,14,15]. It was slightly worse than BP control in the general population in developed countries, in which the prevalence of uncontrolled hypertension oscillates around 67% to 79% [1,28,29,30]. Non-adherence is a well-known factor that influences the effectiveness of hypertension treatment [31,32], and it significantly increases the risk of developing CVD [33]. MDIS was shown in a previous review to worsen therapy adherence and, therefore, potentially worsen hypertension control [8]. However, it was observed only in one study included in the review. There are several factors that are considered hypertension control barriers, and these barriers are categorized into three categories: patient-related, physician-related, and medical environment [31]. Considering patient-related factors, the central one is treatment adherence. Until now, MDIS was not mentioned as a classic patient-related hypertension control barrier, and this should be changed. In this review, MDIS was proven not only to be significantly associated with hypertension control but also to be prevalent in the hypertensive population. Although the studies included in this review did not differentiate if MDIS is a cause of poor hypertension control or it is only an association, MDIS should not be omitted while describing poor control risk factors. Further studies are needed to establish the relationship between MDIS and hypertension control.

### 4.6. Intolerated Drugs

MDIS patients most often experienced ADRs after antibiotics and analgesics (although hypertensive populations were studied, cardiovascular drugs as the reason for MDIS occurred less frequently). These medications slightly differ from those identified in the general population to cause most ADRs in adults (cardiovascular and neurological drugs—including analgesics) [5]. Another systematic review considering the elderly population also found psychotropics, antihypertensives, and analgesics to be involved most often in ADRs [34]. However, one study in this review reported a high prevalence of ADRs after ACE-I, BB, ARB, and CCB. Thus, unambiguous conclusions are hard to make. This phenomenon might indicate that poor hypertension control in MDIS patients might not be caused by simply covert therapy non-adherence, and this hypothesis should be further studied.

Nevertheless, our review’s frequency of drugs causing ADRs is similar to a previously published review about MDIS, which identified antibiotics and non-steroidal anti-inflammatory drugs as the most frequent causes of ADRs [8]. This indicates similarities between MDIS in general and MDIS in the hypertensive population.

### 4.7. Clinical Implications

Our results emphasize that MDIS is not rare among hypertensive patients and significantly influences hypertension control. Physicians should carefully assess ADRs, especially by detailed history taking, avoid unnecessary polypharmacy in cases of suspected intolerance, and recognize MDIS as a potential barrier to achieving target BP values.

### 4.8. Study Limitations

Although the review was conducted with caution, there are several limitations to our study. Firstly, there was considerable heterogeneity among the included studies. We attempted to address this by excluding the study with a different MDIS definition, but other factors, such as varying study designs, may also have contributed. Secondly, the amount of collected data was small, making it difficult to draw definitive conclusions. However, after a thorough search of four databases, it might be assumed that there is no additional data on this subject. Therefore, we aimed to highlight the significance of MDIS and the lack of research on the phenomenon. Finally, due to the high variability and limited number of studies, the results should be interpreted with caution, as the level of certainty is low.

## 5. Conclusions

In conclusion, this review focused on MDIS prevalence and risk factors. MDIS remains inadequately studied, and further research (also in the hypertensive population) is necessary, especially considering its high prevalence of up to 10% (although the high heterogeneity of the included studies limits the estimation). Furthermore, MDIS should be included in classic risk factors of poor hypertension control.

## Figures and Tables

**Table 1:** Characteristics of the included studies.

First Author	Year	Country	Study Design	Number of Patients	Number of Patients with MDIS	Number of Patients Without MDIS	Number of Patients with Single Drug Intolerance	MDIS Definition Included in the Study
Okeahialam	2017	Nigeria	Cross-sectional study	489	15	474	-	Intolerance to ≥3 drugs with no clear immunological mechanism
Polaczyk	2023	Poland	Cross-sectional study	1000	80	920	320	ADRs to ≥3 different classes of drugs
Davies	2003	United Kingdom	Case–control study	233	66	167	52	Intolerance to 2 or more antihypertension drugs
Antoniou	2016	United Kingdom	Case–control study	786	79	707	-	ADRs to ≥3 drugs of any class without a known immunological mechanism

ADRs—adverse drug reactions; MDIS—multiple drug intolerance syndrome.

**Table 2:** Pooled prevalence of ADRs.

ADR	Pooled Prevalence [%]	95% CI for Pooled Prevalence	I^2^ [%]
Electrocyte imbalance	2.50	0.04–7.39	Only one study with this ADR
Hypotension	30.00	20.4–40.55	Only one study with this ADR
Cough	33.94	8.73–64.44	91.42
Edema	38.86	2.95–82.45	95.62
Bradycardia	11.25	5.12–19.23	Only one study with this ADR
Skin lesions	36.71	11.04–66.57	91.09
Gastrointestinal disorders	26.25	19.74–33.32	0
Allergic reactions	66.25	55.47–76.25	Only one study with this ADR
Bleeding	5.00	1.1–11.08	Only one study with this ADR
Abnormal laboratory tests results	1.80	0–5.29	Only one study with this ADR
Muscular pain	4.51	1.09–9.77	0
Headache	71.47	56.5–84.44	32.51
Vasomotor reactions	26.26	0–70.24	89.42
Palpitations	18.77	2.23–43.5	71.05
Sore or dry mouth/throat	7.58	2.2–15.42	Only one study with this ADR
Tiredness	37.11	0–100	95.67
Wheeze	16.57	6.11–30.41	36.37
Dyspnea	12.13	5.19–21.25	Only one study with this ADR
Sleep disturbances	7.64	2.58–14.81	2.51
Urinary frequency	15.16	7.38–24.94	Only one study with this ADR
Gout	12.13	5.19–21.25	Only one study with this outcome
Impotence	4.55	0.58–11.21	Only one study with this ADR
Gynecomastia	3.04	0.05–8.92	Only one study with this ADR
Dizziness	16.26	4.72–32.19	47.35
Bad feeling	59.98	33.86–83.65	Only one study with this ADR
Pain (other than headache)	53.08	0–100	98.46
Tinnitus	20.06	3.05–44.62	Only one study with this ADR

ADR—adverse drug reactions, CI—Confidence Interval.

**Table 3:** MDIS risk factors.

Risk Factor	OR or Difference (Non-MDIS vs. MDIS)			
	Okeahialam	Polaczyk	Antoniou	Davies
Female sex	55% vs. 67%	OR = 2.05 *	13% vs. 40% *	50% vs. 67% *
Older age	-	OR = 1.00	47 vs. 66 years *	59 vs. 64 years *
Disease duration	-	Median: 10 vs. 15 years *	-	-
Gastrointestinal tract diseases	-	OR = 2.33 *	GERD: 42% vs. 10%	-
Psychiatric diseases	-	OR = 1.46	Anxiety disorders: 3% vs. 16%	in text
Rheumatoid diseases	-	OR = 3.14 *	-	-
Endocrine diseases	-	OR = 2.09 *	-	-
Respiratory system diseases	-	OR = 2.14 *	-	-

GERD—gastroesophageal reflux disease, MDIS—multiple drug intolerance syndrome, OR—odds ratio. * statistically significant.

**Table 4:** Prevalence of drug classes intolerance.

Drug Class	Okeahialam	Polaczyk
ACE-I	33.33%	20.00%
BB	73.33%	8.75%
ARB	60.00%	8.75%
CCB	73.33%	15.00%
Diuretics	80.00%	8.75%
Antiplatelets	NR	12.5%
Anticoagulants	NR	3.75%
Statins	NR	8.75%
Antibiotics	NR	46.25%
Analgesics	46.67%	43.75%
Dihydroergotoxine	13.33%	NR
Reserpine	13.33%	NR
Alpha blocker	6.67%	NR
Methyldopa	13.33%	NR
MRA	6.67%	NR

ACE-I—angiotensin converting enzyme inhibitor, ARB—angiotensin receptor blocker, BB—Beta-blocker, CCB—calcium channel blocker, MRA—mineralocorticoid receptor antagonist, NR—not reported.

## Data Availability

The raw data supporting the conclusions of this article will be made available by the authors on request.

## Abbreviations

The following abbreviations are used in this manuscript:

ACE-I	Angiotensin-converting enzyme inhibitors
ADR	Adverse drug reactions
ARB	Angiotensin receptor blockers
BB	Beta-blockers
CCB	Calcium channel blockers
CI	Confidence interval
CVD	Cardiovascular diseases
DBP	Diastolic blood pressure
ESC	European Society of Cardiology
GERD	Gastroesophageal reflux disease
MDIS	Multiple drug intolerance syndrome
PRISMA	Preferred Reporting Items for Systematic Reviews and Meta-Analyses
SBP	Systolic blood pressure
